# Glycosylation status of the C. *albicans* cell wall affects the efficiency of neutrophil phagocytosis and killing but not cytokine signaling

**DOI:** 10.3109/13693786.2010.551425

**Published:** 2011-01-24

**Authors:** Chirag C Sheth, Rebecca Hall, Leanne Lewis, Alistair J P Brown, Frank C Odds, Lars P Erwig, Neil A R Gow

**Affiliations:** The Aberdeen Fungal Group, School of Medical Sciences, Institute of Medical Sciences, University of Aberdeen, Aberdeen, UK

**Keywords:** C. albicans, glycosylation, innate immunity, PMN

## Abstract

The cell wall of the opportunistic human fungal pathogen, *Candida albicans* is a complex, layered network of rigid structural polysaccharides composed of β-glucans and chitin that is covered with a fibrillar matrix of highly glycosylated mannoproteins. Poly-morphonuclear cells (PMNs, neutrophils) are the most prevalent circulating phagocytic leukocyte in peripheral blood and they are pivotal in the clearance of invading fungal cells from tissues. The importance of cell-wall mannans for the recognition and uptake of *C. albicans* by human PMNs was therefore investigated. *N-* and *O*-glycosylation-deficient mutants were attenuated in binding and phagocytosis by PMNs and this was associated with reduced killing of *C. albicans* yeast cells. No differences were found in the production of the respiratory burst enzyme myeloperoxidase (MPO) and the neutrophil chemokine IL-8 in PMNs exposed to control and glycosylation-deficient *C. albicans* strains. Thus, the significant decrease in killing of glycan-deficient *C. albicans* strains by PMNs is a consequence of a marked reduction in phagocytosis rather than changes in the release of inflammatory mediators by PMNs.

## Introduction

*C. albicans* is ordinarily found as a commensal yeast colonizing the human gastrointestinal tract. It is, however, able to invade and cause deep-seated infections in individuals with compromised host defenses arising from trauma, the administration of immunosuppressive agents, a variety of disease and association with a number of genetic mutations that affect immune recognition [1-3]. Invasive, disseminated candidiasis for a range of *Candida* species is associated with mortality rates of 30-60% with *C. albicans* being the most common and pathogenic of the species known to cause invasive disease [4-8]. Many of the virulence traits of this fungus are related to the properties of highly glycosylated proteins in the outer wall and secretome of the organism.

Circulating polymorphonuclear (PMN) cells play a central role in the control mechanism preventing the spread of infection in the host. Fungal cells are recognized and engulfed by these leukocytes, thereby reducing the number of infectious cells in the body [9,10]. Contact between pathogen and PMN also leads to the further activation of the innate immune system via the production of cytokines and chemotactic factors [11]. Cytokines activate PMNs and other cells in the immediate environment, while chemokine production generates a diffusible leukocyte attraction gradient, drawing increasing numbers of immune cells to the site of infection [12]. Stimulated neutrophils activate a programmed antimicrobial response, known as the respiratory burst, leading to the release of significant quantities of toxic oxygen and nitrogen free radicals into the local environment, which can also cause inflammation and damage to the cellular integrity of local tissue [10,13]. The importance of PMNs in host defense is highlighted in studies showing that neutropenic patients [14,15] and those with peroxidase-deflciency are found to be highly susceptible to invasive *C. albicans* infections [16,17].

The primary point of interaction and recognition between the host immune system and *C. albicans* occurs at the interface of the outer cell wall of the fungus and the receptor proteins in the leukocyte membranes. Therefore the outer fungal cell wall has an important role to play in denning the outcomes of such exchanges. The composition and structure of fungal cell wall is highly regulated, but the major components in *C. albicans* consist of a rigid, inner skeletal layer of chitin and interlinked β 1,3- and β 1,6- glucan to which a flexible mannoprotein matrix is attached covalently to β-glucans and chitin predominantly via glycosylphosphatidyinositol (GPI) anchors [11,18,19]. Protein mannosylation occurs in two major forms, *N-* and *O*-linked, and many of the key glycosyl transferases have been highly studied in the model yeast *Saccharomyces cerevisiae* [20-22] and in *C. albicans* [23-26].

**Fig. 1:**
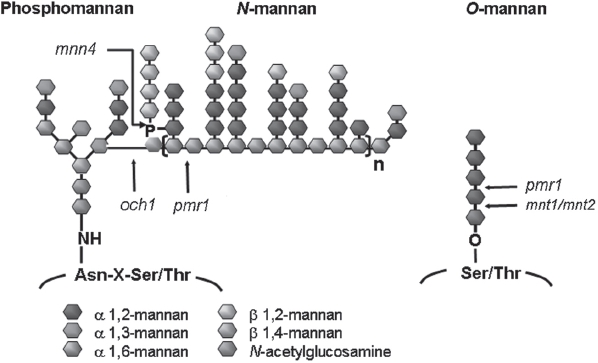
The structure of *N*-mannan (including phosphomannan) and *O*-linked mannan of *C. albicans.* The different glycosyl linkages are shown as different colored hexagons and the point of truncation of the various mutants used in this study is indicated by arrows. The extent of truncation of the *N*-mannan α-1,6 mannose backbone in the *pmr1*Δ mutant is not fixed, and does not remove all of the outer chains, as in the *och1*Δ mutant. See references [23-26].

N-linked mannans are characterized by a conserved [mannose]_8_ [N-acetylglucosamine]2 core structure (numbers in subscript indicating repeating units), appended to more structurally diverse outer chains (Fig. 1). In *C. albicans* the outer chains consist of a α-(1,6)- mannose backbone to which are attached side chains consisting of α-(1,2)-β-(1,2)- and α-(1,3)- linked mannose and phosphoman-nose branches [27]. *C. albicans* N-linked and *O*-linked mannans have been shown to be important for adhesion and other virulence traits and for the induction of the innate immune response in the host, including the production of pro-inflammatory cytokines and anti-mannan antibodies via the stimulation of a number of immune cell types [28-30].

*C. albicans* O-linked mannans are simpler structures, consisting of a linear (unbranched) chain of mannose residues, predominantly α-(1,2)-linked (Fig. 1) [26,31]. *O*-linked mannans are attached to cell wall proteins via serine or threonine residues that are frequently clustered in domains that correspond to the rod-like stems of glycopro-teins that present a globular N terminus towards the extracellular face of the wall [22]. *C. albicans* strains deficient in *O*-mannan synthesis were found to be attenuated in virulence [26,32] and adhesion to epithelial cells [33,34], displayed altered cytokine stimulation profiles and were less sensitive to killing by neutrophils [35].

To investigate the importance of *C. albicans* cell wall *N-* and *O*-linked glycosylation on the fungus-human PMN interaction, we used a series of *C. albicans* strains defective in different cell wall glycosylation components. The host-fungus interaction was characterized by quantifying the proportions of *C. albicans* bound or engulfed by the PMNs, the percentage of fungal cells killed following co-incubation, and PMN activation by measurement of supernatant myeloperoxidase and IL-8. Additionally, the sensitivity of *C. albicans* glycosylation-defective strains to PMN-derived anti-microbial compounds was investigated.

## Materials and methods

### Strains, media and culture conditions

All strains used in this study are listed in Table 1. Strains were maintained as frozen glycerol stocks and grown in Sabouraud broth (1% mycological peptone, 4% glucose) for 16 h at 30°C with shaking at 200 rpm. For the preparation of co-incubation inocula, above cultures were diluted 1:100 into 50 ml fresh sterile Sabouraud broth and incubated at 30°C with shaking at 200 rpm until the OD_600_ reached 0.6. The cells were then washed three times in sterile phosphate-buffered saline (PBS, pH 7.5), and resuspended to a final concentration of 1 × 10^8^ cells/ ml in PBS. Prior to use, an aliquot of the required number of cells was labeled with 0.1 mg/ml fluorescein 5-iso-thiocyanate (F2502, Sigma-Aldrich, UK) in 0.05 M carbonate-bicarbonate buffer (C3041, Sigma-Aldrich, UK), for 15 min at room temperature with continual agitation. Cells were then washed three times with sterile PBS and used immediately in experiments with *C. albicans* cells.

**Table 1:** Candida albicans strains used in this study.

Strain description	Genotype	Strain #	Reference
CAI-4 + CIp10	*Δura3::imm434/Δ ura3::imm434, RPS1/rps1Δ::*CIp10	NGY152	[24]
*och1*Δ	As NGY152 but *och1Δ::hisG/och1Δ::hisG, RPS1/rps1Δ::*CIp10	NGY357	[23]
*och1*Δ + CIp10-*OCH1*	As NGY152 but *och1Δ::hisG/och1Δ::hisG, RPS1/rps1Δ::*CIp10-OCH1	NGY358	[23]
*pmr1*Δ	As NGY152 but *pmr1Δ::hisG/pmr1Δ::hisG, RPS1/rps1Δ::*CIp10	NGY355	[38]
*pmr1*Δ + CIp10-*PMR1*	As NGY152 but *pmr1Δ::hisG/pmr1Δ::hisG, RPS1/rps1Δ::*CIp10-PMR1	NGY356	[38]
*mnt1*Δ/*mnt*2Δ	As NGY152 but *mnt1-mnt2Δ::hisG/mnt1-mnt2Δ::hisG-URA3-hisG*	NGY111	[26]
*mnt1*Δ/*mnt2*Δ + CIp10-*MNT1*	As NGY152 but *mnt1-mnt2Δ::hisG/mnt1-mnt2Δ::hisG, RPS1/rps1Δ::*CIp10-*MNT1*	NGY335	[26]
*mnn4*Δ	As *NGY152 but mnn4Δ::hisG/mnn4Δ::hisG, RPS10::URA3*	CDH15	[24]
*mnn4*Δ + CIp10-*MNN4*n	As *NGY152 but mnn4Δ::hisG/mnn4Δ::hisG, RPS10::CIp10-MNN4-URA3] n*	CDH13	[24]
A9 (Serotype B)	—	—	[39]

### Preparation of human PMNs

Human PMNs were isolated from freshly isolated, EDTA-anticoagulated peripheral venous blood of healthy human volunteers [36]. Briefly, whole blood was diluted 1:2 in sterile PBS and 35 ml aliquots dispensed into 50 ml polypropylene tubes. Gently, 15 ml of Ficoll-Paque PLUS (17-1440-02, GE Healthcare, UK) was layered under the whole blood by sterile Pasteur pipette. Tubes were cen-trifuged at 400 g for 20 min at room temperature. The overlying plasma layer was carefully aspirated and cen-trifuged at 1500 *g* to generate platelet-poor plasma (PPP). The lymphocyte and Ficoll-Paque PLUS layers were aspirated and discarded. The pellet, containing erythro-cytes and PMNs was lysed using RBC lysis buffer (420301, Biolegend, USA) according to the manufacturer's instructions. The resulting PMN pellet was washed twice in sterile PBS at room temperature, with centrifugation at 400 *g* and finally resuspended in pre-warmed RPMI-1640 medium supplemented with 10% donor's PPP. Neutro-phils were examined by light microscopy and cell viability was established as >98% by Trypan Blue dye exclusion [37]. All tests were completed within 5 h of PMN isolation.

As required aliquots of 1 × 10^6^ PMNs were labeled with anti-CD13.PE-Cy5 (A07763, Beckman Coulter, USA) according to the manufacturer's instructions for 30 min at room temperature in the dark. After two washes in sterile PBS with centrifugation at 400 g the cells were ready for use.

### Phagocytosis assay

A sample of 3 × 10^6^ washed FITC-labeled *C. albicans* cells were co-incubated with 1 × 10^6^ freshly isolated, anti-CD13. PE-Cy5 labeled human PMNs (effector: target ratio 3:1), and co-incubated in RPMI-1640 medium supplemented with 10% donor's own serum for 30 min at 37°C with continuous shaking. Cells were harvested by centrifugation at 500 *g,* resuspended and fixed in 500 μl ice-cold PBS with 40 mM EDTA and 1% (v/v) paraformaldehyde, and stored on ice.

### Killing assay

Samples of 3 × 10^6^ FITC-labeled *C. albicans* cells were co-incubated with 1 × 10^6^ freshly isolated, anti-CD13. PE-Cy5 labeled human PMNs (effector: target ratio 3:1), and co-incubated in RPMI-1640 medium supplemented with 10% donor serum for 150 min at 37°C with continuous shaking. No hypha formation was observed inside the neutrophils by this time, thus no killing of neutrophils occurred due to hypha formation within the phagolyso-some. Fungal cells were harvested by centrifugation at 1500 *g,* and the supernatant discarded. Hypotonic lysis of the PMNs (pure water, 1 min at room temperature) was followed by three washes in sterile PBS. Finally the released *C. albicans* cells were resuspended in 1% paraformaldehyde in PBS, containing 4 μg/ml propidium iodide (PI). The percentages of dead cells with PI-stained nuclei were quantified by fluorescence microscopy. A minimum of 3 groups of 100 cells were counted for each strain and the experiment repeated for a total of six independent biological replicates. Controls in the absence of PMNs showed 0% killing, therefore all killing was ascribed to the activities of the PMNs. For fluorescence images on PMNs with attached and phagocytosed *C. albicans* cells a Zeiss Axioplan 2 microscope (63 × magnification) with the Hamanatsu C4742-95 digital camera was used and images were analyzed with Openlab software V4.0.4.

### Flow cytometry analysis of phagocytosis

Flow cytometry analysis of the samples was carried out using a LSRII flow cytometer (Becton Dickinson, Heidelberg, Germany), with computer-assisted evaluation of the generated data by FACS Diva software (Becton Dickinson, Heidelberg, Germany). The fungal cell wall dye Calcofluor White (CFW) was added to a final concentration of 10 μg/ml immediately prior to flow cytometry, allowing distinction between bound and engulfed *C. albicans* cells. In principle, internalized fungal cells are encapsulated by the intact cell membrane of the PMN, thereby preventing their staining by CFW. Free, suspended *C. albicans* cells or cells adhered to the outside of the neutrophil are stained by the fluorophore and were detected via the Pacific Blue channel.

### Cytokine and chemokine assays for PMN activation

Supernatants collected following co-incubation of PMNs and *C. albicans* cells were assayed for markers of PMN activation. Simultaneous screening of 36 cytokines, chemokines and other secretory proteins was carried out using a human cytokine profiler kit (R&D Systems UK, Cat# ARY005) according to the manufacturer's instructions. The cytokine profiler data, in addition to conventional sandwich ELISA-based analysis confirmed evidence from other groups showing that activated neu-trophils release the respiratory burst associated proteins myeloperoxidase and lactotransferrin (LTF) into the environment when stimulated [10]. IL-8 secretion was found to be a consistent marker of PMN activation in this study. Quantification of IL-8 and MPO markers was carried out according to the manufacturer's instructions using commercially available sandwich ELISA-based assays (IL-8 - R&D Systems, Cat# DY208, MPO -Calbiochem, Cat# 475918).

## Results

### *Flow cytometric analysis of attachment and phagocytosis of* C. albicans *by PMN cells*

To characterize the effects of changing glycosylation status upon host: fungus interaction, we challenged purified human PMNs with a range of glycosylation-defective strains of *C. albicans.* Incubations were carried out for 30 min to evaluate glycosylation effects on adherence and phagocytosis. In the following description, we take 'association' to mean all *Candida* cells co-sorting with PMNs following the incubation period (including adhered and phagocytosed subsets), whilst 'attached' or 'adhered' cells refer to subsets of the associated cells bound to the PMN cell surface, undergoing phagocytosis. Various subsets of *C. albicans* yeast cells that were interacting with PMNs were distinguished in the analysis. An 'association' indicates *C. albicans* cells that co-sort with PMNs and includes adhered and phagocytosed yeast cells. Yeast cells that were 'attached' or 'adhered' refers to subsets of associated cells that were bound to the PMN cell surface but were not phagocytosed while 'engulfed' refers to *C. albicans* cells that had been phagocytosed. The strains used in this study are shown in Table 1 and in Fig. 1. Briefly, the *och1Δ* strain lacks the branched, outer part of the *N*-glycan structure [23], the *pmr1* Δ strain has a truncated *O*-glycan and *N*-glycan structure [38], the *mnt1Δ/mnt2Δ* strain contains only a single *O*-linked mannose residue [26] and the *mnn4Δ* strain lacks cell wall phosphomannan including the acid-labile β-1,2 mannan fraction [24]. *C. albicans* serotype B strain A9 differs from the serotype A strains in being deficient in β-1,2 mannose in the acid stable component of the *N*-linked mannan [39]. Serotype A strains include the SC5314 strain that is the genetic background for all the mutants used in this study.

**Fig. 2:**
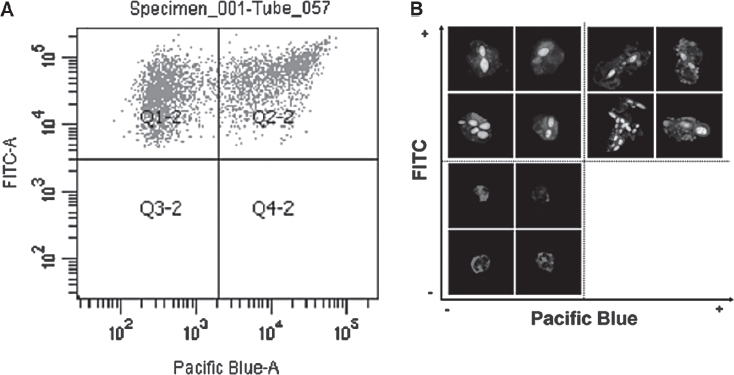
FACS assay for binding and phagocytosis and validation confocal fluorescence microscopy. Test co-incubation samples of *C. albicans* and PMNs (ratio 3:1) were analyzed by FACS (SI Materials and Methods - online only). (A) A scatter-graph of PMN associated-*C. albicans* cells plotted as a function of FITC versus CFW fluorescence intensities. Two sub-populations of fungal cells are resolved, corresponding to engulfed (quadrant Q1-2) and adhered (quadrant Q2-2) *C. albicans* cells. PMNs not associated with *C. albicans* cells were excluded from the analysis by FACS software. (B) Confocal fluorescence microscopic images of representative sorted cells from quadrants Q1-2 and Q2-2 validating the FACS analysis and gating strategy. Engulfed, FITC-labeled *C. albicans* cells are visible in the upper-left quadrant, whilst PMNs with adhered fungal cells stained with the cell-wall specific dye CFW are shown in the upper-right quadrant. PMNs not associated with *C. albicans* cells were sorted and visualized (lower-left quadrant), thereby validating the selection of the PMN-specific antibody.

Evaluation of FACS assay that measured the binding and uptake of *C. albicans* by neutrophils was also validated by fluorescence microscopy. Two sub-populations of *C. albicans* cells could be distinguished using FACS (Fig. 2A). *C. albicans* cells association with PMNs was expressed as a function of FITC fluorescence versus CFW fluorescence (measured on the Pacific Blue channel). *C. albicans* cells in quadrant Q1-2 (FITC +, CFW-) represent engulfed cells whilst those in quadrant Q2-2 (FITC +, CFW +) represent cells attached to the PMN surface (Fig. 2A). Engulfed and adhered (non-engulfed) cells from each of the sub-populations were also visualized by fluorescence microscopy (Fig. 2B) and corresponded to cells within the various FACS quadrants. Empty PMNs were excluded from the plot in Fig. 2A, however they were visualized by fluorescence microscopy demonstrating positive α-CD13 antibody selection of the target immune cell type (Fig. 2B). These data together demonstrate the robustness of the FACS technique in characterizing adherence and phagocytosis of *C. albicans* cells by human PMNs.

**Fig. 3:**
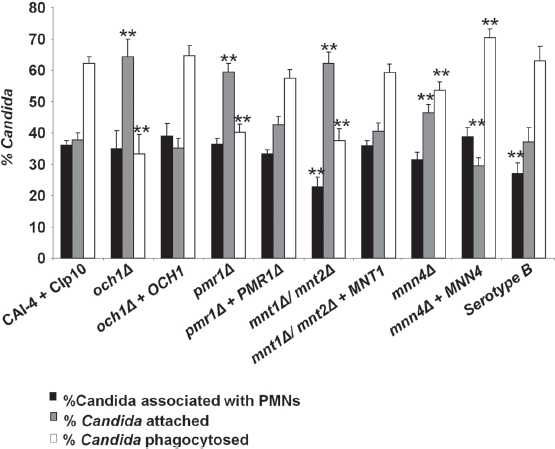
Analysis of binding and phagocytosis of *C. albicans* glycosylation-deficient mutant strains by FACS. Bars indicate the percentage of *C. albicans* cells associated with, bound to, or engulfed by human PMNs following 30 min of co-incubation at 37°C. The solid bars represent the percentage of *C. albicans* yeast cells (as a proportion of the total *C. albicans* cells counted) co-sorting with PMNs. This reflects differences in the recognition and binding of the various *C. albicans* strains by PMNs. Grey bars show the percentage of *C. albicans* yeast cells adhered to versus those engulfed by PMNs (subsets of the *C. albicans* yeast cells co-sorted with PMNs) and represent a measure of the relative ability of PMNs to phagocytose the *C. albicans* yeast cells. The open bars represent the proportion of associated cells that had been phagocytosed at this time point. Plotted values are the mean ± standard error of triplicate experiments using PMNs from six independent donors. Statistical significance was carried out by a student's t-test (vs the value for the control strain in each case): **P < 0.05.

Flow cytometry was used to investigate the adherence and engulfment of *C. albicans* cells by human PMN following co-incubation for 30 min at 37°C. The gating strategy employed to analyze the flow cytometry data is described in the Online Supplementary Information (SI): Materials and Methods. After 30 min co-incubation, approximately 36 ± 2% (mean ± SE - and in all cases below) of the control *C. albicans* cells were co-sorted with PMNs (Fig. 3). The *mnt1Δ/mnt2Δ* and *C. albicans* Serotype B strains, had reduced association with PMN cells (23 ± 3 and 27 ± 3%, [*P* < 0.01 and *P* < 0.05] respectively) suggesting that *O*-mannan or β 1,2 linked mannan was implicated in PMN recognition and binding. PMN cells were attenuated in their ability to engulf *N*- and *O*-glycosylation-defective *C. albicans* strains (Fig. 3). Analysis of the flow cytometry data showed that 62 ± 2% of the cells of the CAI-4 + CIp 10 control strain bound to PMNs were engulfed within 30 min co-incubation. In contrast, only 33 ± 6% of *och1Δ,* 40 ± 3% of *pmr1* Δ, and 38 ± 4% of *mnt1Δ/mnt2Δ* cells were engulfed *(P <* 0.01) (Fig. 3). Consequently, the reduced engulfment of mutant strains resulted in an accumulation of attached (adhered), non-engulfed *C. albicans* cells at the PMN surface. In turn the percentage of adhered *C. albicans* cells rose from 38 ± 2% in the control (CAI-4 + CIp10) strain to 64 ± 6% *(och1Δ),* 60 ± 3% (pmr1Δ), and 62 ± 4% *(mnt1Δ/mnt2Δ)* for the glycosylation-defective mutants respectively. Complementation of the deleted genes in all cases reverted the ratio of engulfed:adhered *C. albicans* to that resembling the control strain. The *mnn4Δ* strain lacking *N*-linked cell wall phosphomannan exhibited a relatively minor reduction in engulfment (53 ± 3%) in comparison to the other strains tested, although this was still significantly less than the control strain (Fig. 3). Likewise, the percentage of mnn4Δ cells adhered to the PMNs increased slightly but significantly with respect to the control strain (up to 46 ± 3%). Complementation of the mutant with the wild type gene restored the control profile of engulfed and adhered *C. albicans* cells to control levels although the mnn4Δ reintegrant strain, which contained multiple *MNN4* alleles was more readily engulfed than the wild type (Fig. 3). The *C. albicans* Serotype B strain that lacked acid-stable (3 1,2-linked mannose residues in the *N*-glycan outer chain had little difference in adherence and uptake into neutrophils (Fig. 3). Therefore, the cell wall glycosylation status (alterations in *N-* and *O*-linked glycosylation and phosphomannosylation) but not changes in the β 1,2 mannose content, reduced the engulfment of adherent fungal cells by human PMNs.

**Fig. 4:**
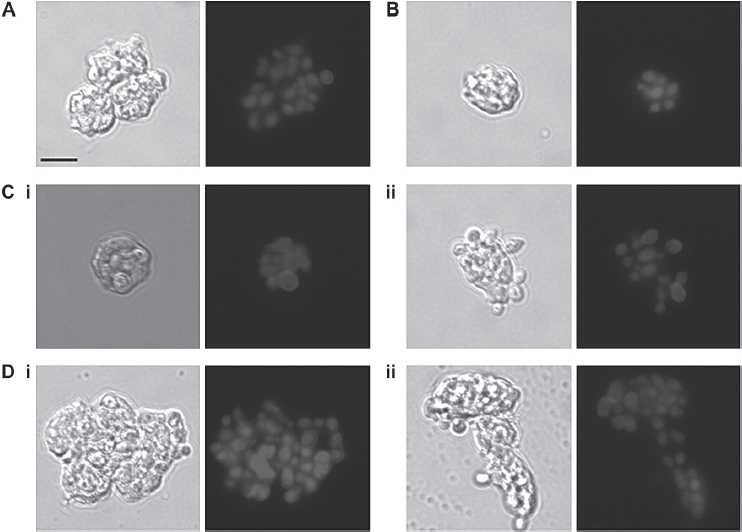
Glycosylation status of the *C. albicans* cell wall is important for phagocytosis by neutrophils. FITC-labeled *C. albicans* cells (3 × 10^6^) were incubated with purified anti-CD13-PE-Cy5 labeled neutrophils (1 × 10^6^) at 37°C for 30 minutes in RPMI medium supplemented with 10% donor's plasma. Cells were fixed with 1% paraformaldehyde and 40mM EDTA and 10 μg/ml Calcofluor White added 1 minute prior to microscopy. (A) CAM + CIp10, (B) Serotype B, (C) (i) Δ*mnn4* (ii) Δ*mnn4 + MNN4,* (D) (i) Δ*och1* (ii) Δ*och1* + *OCH1*. Note in (D) (i) that the FITC-stained yeast cells have a blue Calcofluor White-stained halo indicating they are on the PMN surface, while in (D) (ii) the FITC-stained cells are phagocytosed and do not stain with Calcofluor White. Scale bar represents 10 μm.

The effect of glycosylation status of *C. albicans* phagocytosis by purified anti CD13-PE-CY5-labeled human PMNs was validated independently by microscopy. PMNs were co-incubated with FITC-labeled *C. albicans* glycosylation mutants. After fixation, the *C. albicans* cell wall dye, Calcofluor White, was added immediately prior to microscopy to distinguish between *C. albicans* cells adhered to the PMN surface and those that had been internalized through phagocytosis. Prolonged exposure to the fixation solution (1% paraformaldehyde, 40 mM EDTA) resulted in non-specific staining of the PMN nuclei by Calcofluor White, presumably due to increased permeability of the cell membrane. Therefore, to ensure that any non-specific staining did not interfere with FACS analysis, samples were analyzed minutes after fixation when no nonspecific CFW staining was observed. The majority of the CAI4-CIp10 (wild type control strain) cells associated with PMNs fluoresced only under the FITC filter, suggesting that they had been phagocytosed and were not exposed on the PMN surface. However, the *C. albicans* strains that were defective in cell wall glycosylation fluoresced under both the FITC and Pacific Blue channels, indicating that the majority of *C. albicans* cells had adhered to the PMN surface, but that the PMNs were unable to phagocytose the cells. Reintroduction of a single copy of the desired gene again restored the ability of the PMNs to phagocytose the *C. albicans* strain to almost wild type levels, confirming that the glycosylation status of *C. albicans* is important for phagocytosis (Fig. 4).

### *Comparison of killing of* C. albicans *glycosylation-defective mutant strains by PMN cells*

Next we assessed whether changes in the fungal cell-wall glycosylation status altered susceptibility to killing by PMNs. The sensitivity of *C. albicans* cell-wall glycosyla-tion-deficient strains to PMN-mediated damage was investigated using phagocytosis-dependent and phagocytosis-independent killing assays. For the former, *C. albicans* cells were subjected to an extended co-incubation period of 150 min to assay the killing efficiency of PMNs with respect to fungal glycosylation status. Analysis of pro-pidium iodide stained fungal cells showed that 66 ± 4% of the control fungal cells were killed in this period (Fig. 4). Glycosylation defects reduced the proportion of fungal cells killed by PMNs. Only 46 ± 4% of och1Δ, 51 ± 4% of *pmr1Δ,* 41 ± 4 % of *mnt1Δ/mnt2Δ* and 46 ± 4% of *mnn4Δ* cells were killed *(P <* 0.05 in all cases, except forpmr1Δ strain, where *P =* 0.15) (Fig. 4). Complementation of the deleted genes restored some measure of susceptibility to killing, although this was not always to control levels. A positive correlation existed between the percentage of dead *C. albicans* cells and the percentage of cells that had been engulfed (Pearson's correlation coefficient, [r] =+0.71).

### *Effects of glycosylation upon phagocytosis-independent killing of* C. albicans *by PMNs*

PMNs can kill *C. albicans* by several mechanisms and reciprocally the ability of *C. albicans* to reduce PMN viability may be affected by the presence of other microorganisms and antigens. PMNs can ingest and digest pathogens but also release of large quantities of enzymes and toxic free-radicals into the environment, causing damage and death to non-ingested pathogens in the vicinity of the immune cells [10]. Here we investigated the viability of PMNs in the presence and absence of bacterial cells or bacterial LPS and the effect of PMN super-natants on the viability of *C. albicans.*

First, we investigated the effect of 30 min co-incubation with LPS, two bacterial and one *C. albicans* glycosylation-deficient strain on PMN viability by Trypan Blue exclusion. Stimulation by LPS, heat-killed *Pseudomonas aeruginosa* and heat-killed *Escherichia coli* resulted in a higher proportion of dead PMNs (58 ± 10%, 40 ± 11% and 45 ± 5% respectively), as compared to pre-stimulation with any of the *C. albicans* strains CAI-4 (28 ± *3%),pmr1Δ* (29 ± 1%) and *pmr1* Δ + *PMR1* (37 ± 7%) respectively (SI Fig. S2 - online only). Therefore, more PMNs were killed following co-incubation with bacterial cells and LPS as compared to following co-incubation with the *C. albicans* strains tested.

**Fig. 5:**
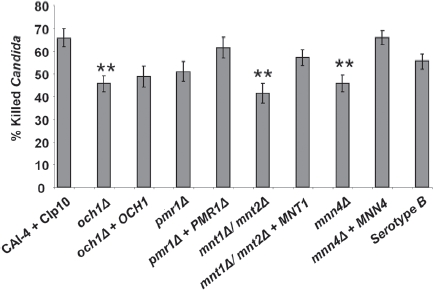
*C. albicans* killing assay. Bars show the percentage of the tested *C. albicans* killed following 150 min co-incubation with PMNs at 37°C. Following incubation, PMNs were lysed by hypotonic shock. The remaining *C. albicans* were washed and stained with 4 μg/ml propidium iodide to stain exposed nucleic acid in dead and terminally damaged cells. A minimum of 100 cells of each strain were counted and the percentage of dead (PI+) cells calculated. Plotted values are the mean ± standard error of six experiments with independent donors, in which each combination was tested in triplicate. Statistical significance was carried out by a student's t-test (vs the value for the control strain in each case): **P < 0.05.

Next, we investigated whether PMNs exposure to wild-type and *C. albicans* mutant strains would affect the composition and quantity of microbicidal compounds released into the cell-culture supernatant (Supplementary Materials and Methods: Supplementary method 2 - online only). The results in SI Table S1 indicate that following the 30 min pre-incubation period, fungal cells generally elicited higher levels (20-25 ng/ml) of MPO production by PMNs as compared to LPS (9 ± 1 ng/ml), *P. aeruginosa* (13 ± 2 ng/ml) and *E. coli* (12 ± 4 ng/ml). IL-8 induction was similar irrespective of the stimulus (∼16 ± 2 ng/ml) Fig. 5). The efficacy of these cell-culture supernatants to kill *C. albicans* was characterized by co-incubation with fungal cells over 150 min.

The percentage of dead *C. albicans* cells following 150 min incubation with cell culture supernatants from pre-stimu-lated PMNs showed no statistically significant difference, with respect to the pre-stimulant. All the *C. albicans* strains tested in this assay were equally sensitive to supernatant from pre-stimulated PMNs (42 ± 8% killed *C. albicans* cells) (SI Fig. S2 - online only). Supernatant concentrations of MPO and IL-8 did not change during co-incubation with fungal cells (SI Table S1 - online only). Yeast strains of the *pmr1Δ* mutant showed similar sensitivity to cell damaging agents released by activated neutrophils when compared to their wildtype control.

### PMN stimulation by cytokine and chemokine analysis

Activated PMNs are able to secrete a variety of cytokines and chemokines into their immediate environment, resulting in autoinduction and the generation of chemoattrac-tant gradients. The activation of PMNs in response to stimulation by glycosylation-defective *C. albicans* strains was characterized by quantifying supernatant levels of secreted markers of activation. Initial testing using the R&D Systems proteome profiler cytokine assay kit (R&D Systems UK, catalog # ARY005), which has been effective in the analysis of cytokine release from monocytes [40] and T-cells [41], identified IL-8, MPO and LTF as being upregulated. Supernatant levels of IL-8, IL13, mac-rophage migration inhibitory factor (MIF), macrophage inflammatory protein-1α (MIP1α), granulocyte colony stimulating factor (G-CSF), granulocyte-macrophage colony stimulating factor (GM-CSF) and complement component C5a, were found to be elevated with respect to the control, with C5a, IL-8 and MIF among the highest induced (not shown).

**Fig. 6:**
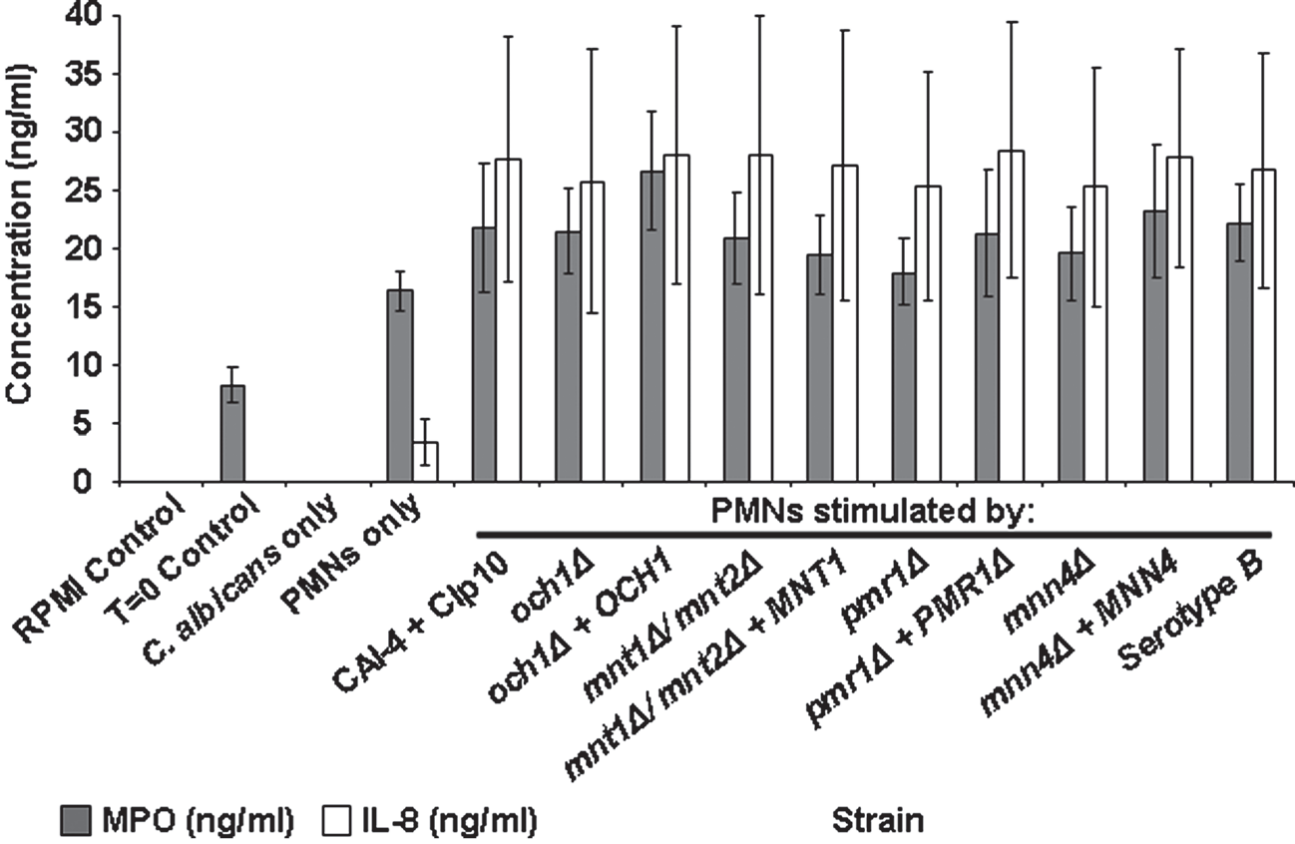
Myeloperoxidase and IL-8 quantification in *C. albicans*-PMN supernatants. The plot shows MPO and IL-8 production in cell culture supernatant following the co-incubation of PMNs (10^6^ cells) with *C. albicans* strains (3 × 10^6^ cells) for 30 min at 37°C. MPO and IL-8 concentrations were determined by ELISA assay kits. Controls were cell culture supernatant (RPMI only), pre-incubation supernatant (T = 0 control), and cell culture medium from samples lacking either PMNs (*C. albicans* only) or *C. albicans* cells (PMNs only). Plotted values are the mean ± standard error of six experiments with independent donors, in which each combination was tested in triplicate.

PMN cell activation was characterized by investigating the production of myeloperoxidase (MPO) and the cytokine IL-8 (CXCL8) in cell culture supernatant following 30 min co-incubation with *C. albicans* strains (LTF was not studied in detail as similar to MPO it is a marker of the respiratory burst). MPO catalyses the formation of hypochlorous acid and other toxic reactive oxidants following neutrophil activation, and has been shown to play a key role in the control of *C. albicans* infection by PMNs [17,42,43]. IL-8 is produced by a variety of human immune cells including neutrophils, and is a potent stimulant of PMN cells [44,45]. There was a basal level of MPO production (8 ± 2 ng/ml) following PMN purification, as evidenced by the T = 0 sample (Fig. 6).

Analysis of the cell culture supernatant of PMNs that had not been exposed to *C. albicans* resulted in a basal level of MPO production (16 ± 2 ng/ml). Co-incubation with *C. albicans* resulted in increased levels of MPO with a mean of 22 ± 5 ng/ml. There was no significant difference in the induction of MPO production between the glycosylation-deficient and parent strains of *C. albicans* tested (Fig. 6). Following co-incubation of PMNs with *C. albicans* super-natants were assayed and found to contain higher levels of IL-8 (an average of 27 ± 11 ng/ml across the strains tested). However, no significant differences in IL-8 production were found between the mutant and wildtype strains that were tested. Thus PMNs upon exposure to *C. albicans* produced MPO and IL-8, but this was not dependent on the glycosy-lation status of the cell wall.

## Discussion

*C. albicans* is the most common cause of invasive noso-comial disease [46,47]. Increased mortality rates and failure to clear infection in neutropenic patients [48] and those with genetic abnormalities related to neutrophil function [49] highlight the critical role played by PMNs in controlling the growth and spread of pathogens. In this study we examined the effects of *C. albicans* cell wall glycosylation on the binding and phagocytosis by PMNs. We show that *O*-glycosylation plays a role in the initial association between PMNs and *Candida* as *O*-glycan-deficiency resulted in significantly fewer cells associated with PMNs as compared to control and *N-*glycosylation mutant strains. In addition, loss of cell wall *N-* and *O*-glycans results in a reduced capacity of PMNs to phagocytose fungal cells. PMNs were able to phago-cytose significantly fewer *och1Δ, pmr1Δ,* and *mnt1Δ/ mnt2Δ* mutant cells as compared to the control strain. Binding of these mutants was not abolished however, implying that residual cell wall components such as β-glucans also play a role in uptake by PMNs. In such strains a corresponding increase in the percentage of adhered cells was found reflecting the disruption of the engulfment process leading to an accumulation of *C. albicans* on the PMN cell surface. The data suggest that recognition and uptake of fungal cells by PMNs is a two-step process and suggests that independent receptor-ligand interaction mediated by cell surface mannans may be required, for each stage to be successfully completed. This is in keeping with previous observations for the phagocytosis of apoptotic cells where a two step process for successful phagocytosis has been proposed [36].

Previous work from our laboratory has shown that loss of cell wall phosphomannan had little impact upon *C. albicans* virulence in a murine model [24]. This study advances the body of information relating to the role of phosphomannan at the fungal cell surface, showing that mutants lacking the epitope are slightly attenuated in their phagocytosis by PMNs. The decrease in phagocytosis was observed to be much greater in *N-* and *O-*glycan-deficient mutants as compared to the mnn4Δ strain indicating the relatively minor role of phosphomannan as compared to glycosylation, in the recognition, attachment and phagocytosis by neutrophils. Therefore non-phagocytosis-dependent interactions and adhesion to PMNs is unlikely to be due to charge interactions with PMN in the *C. albicans* cell wall. This highlights important differences in the phagocytosis of *C. albicans* between macrophages and PMNs as recent data shows that loss of phosphomannan content profoundly reduces the ability of macrophages to recognize and ingest *C. albicans* whereas loss of *O*-mannan enhances phagocytosis [50]. Loss of mannan also results in reduced cytokine production by monocytes [2,51]. The percentage of cells of *Candida albicans* adhered to and phagocytosed by PMNs was not significantly different in the serotype A and serotype B strains. The serotype B strain bears cell wall mannans containing β1, 2 mannose residues, recently shown to be associated with both *N* and *O* mannans [52]. This suggests that β1, 2 mannose residues and their putative ligand, galectin-3 do not appear to be required for PMN binding, phagocytosis or killing.

The dependence of the processes of recognition, phagocytosis and killing of *C. albicans* by PMN cells upon specific receptor-ligand combinations have been described previously. Lavigne *et al.* showed that fungal cell wall β-glucan is important for the recognition of adhered *C. albicans* cells, and is able to stimulate the antimicrobial respiratory burst in PMNs [53]. The importance of complement binding to the *C. albicans* cell surface was described by Yamamura *et al.,* who showed that complement factor C3 was required for *C. albicans* killing, but not phagocytosis [54]. Work by Taylor *et al.* links these two studies by showing that murine dectin-1 (receptor for fungal P-glucans)-deficient neutrophils were found to be impaired in their ability to bind un-opsonized zymosan particles, resulting in a lowered respiratory burst [55]. The use of opsonized zymosan particles or *C. albicans* cells restored binding, however the dectin-1 deficient PMNs remained attenuated in their ability to generate the respiratory burst. Dectin-1 deficiency resulted in a strong decrease in the ability of PMNs to kill unopsonized *C. albicans* cells, however, killing was restored with opsonized *C. albicans* [54]. Purified soluble *C. albicans* cell wall mannan has also been shown to inhibit the interaction between *C. albicans* cells and human PMNs, demonstrating the requirement for glycans during attachment, and supports the data from our study [56]. Studies in mice infected with *C. albicans* showed that TLR2 expression on neutrophils was required for efficient recognition of and chemotaxis towards fungal cells. Additionally, neutrophils from TLR2-/- mice were shown to be attenuated in their phagocytic activity towards *C. albicans* cells as well as expressing lower levels of nitric oxide and myeloperoxidase [57].

There were no significant differences in the levels of MPO or IL-8 produced by PMNs in the first 30 min following contact with the *C. albicans* glycosylation mutants tested in our study. Our data confirmed that contact with fungal cells results in activation-dependent secretion of MPO and IL-8 into the culture medium [16,42,58,59]. Co-incubation of *C. albicans* cells with cell culture supernatant from pre-stimulated PMNs showed that: (i) there was no significant difference between the levels of MPO and IL-8 induced by the control strain and the pmr1Δ glycosylation-deficient mutant; and (ii) there was no significant difference in the percentage of *C. albicans* killed between the control and glycosylation-deficient strain. These data together suggest that PMNs at least in terms of their secretory profile do not respond specifically to the cell wall glycans of *C. albicans* cells, instead generating a broad non-specific cellular response to the fungus.

This study is the first to show that ubiquitous cell wall *N-* and *O*-linked glycans are involved in the recognition, binding and phagocytosis of *C. albicans* by human PMNs. Interestingly, studies using human peripheral blood mono-nuclear cells have found that *N*-linked glycans are predominantly recognized by the mannose receptor whilst *O*-glycans bound TLR4 and that these interactions are important for cytokine-stimulating activity by yeast cells [60]. Here we show that the production of chemokines and respiratory burst enzymes by PMNs appears not to be dependent on cell wall mannosylation.
