# Supplementary material for: Glycosylation status of the C. albicans cell wall affects the efficiency of neutrophil phagocytosis and killing but not cytokine signaling
Source: Med Mycol. 2011 Jan 24;49(5):513–24. doi: 10.3109/13693786.2010.551425 (PMC3119872; doi:10.3109/13693786.2010.551425)
Supplement: Supplementary information [file tmmy49-513supp.pdf]

Supplementary material for Sheth CC, Hall R, Lewis L, Brown AJP, Odds FC, Erwig LP & Gow NAR. Glycosylation status of the *C. albicans* cell wall affects the efficiency of neutrophil phagocytosis and killing but not cytokine signalling, *Medical Mycology*, 2011; **49**: 513–524.

Supplementary Information: Materials and Methods

Supplementary Methods 1: Flow Cytometry Gating Strategy

Polymorphonuclear cells (PMNs) and *Candida albicans* samples following co-incubation were analyzed by flow cytometry. The sub-population of PMNs was defined by electronically selecting (gating) the population according to their forward- and side-scatter characteristics (Fig. S1A). The population was further refined by back-gating from a second scatter plot (Fig. S1B) of side-scatter versus signal from the PerCP channel (used to monitor the PE-Cy5 signal from the labeled PMN cells). Having identified the labeled PMN cell population, the FITC-positive *C. albicans* population was visualized and gated on a simple side-scatter versus FITC-channel plot (Fig. S1C). The population of cells positive for CFW staining were defined by a gate placed on a side-scatter versus Pacific Blue channel (used for capturing the CFW signal) scatter plot (Fig. S1D). The FITC-positive population in the sample was interrogated by plotting events from the FITC-positive gate (from Fig. S1C) on a side-scatter versus forward scatter plot (Fig. S1E). Using this technique, it was possible to identify the population of FITC-positive *C. albicans* cells (shown bound by gate P3, Fig. S1E) directly interacting with the PMN population. The effectiveness of this analysis has been demonstrated by showing the interacting population P3 in all the panels of Fig. S1. Population P3 is clearly comprised of PMN cells and *C. albicans*, and is split in Fig. S2D, indicating that both

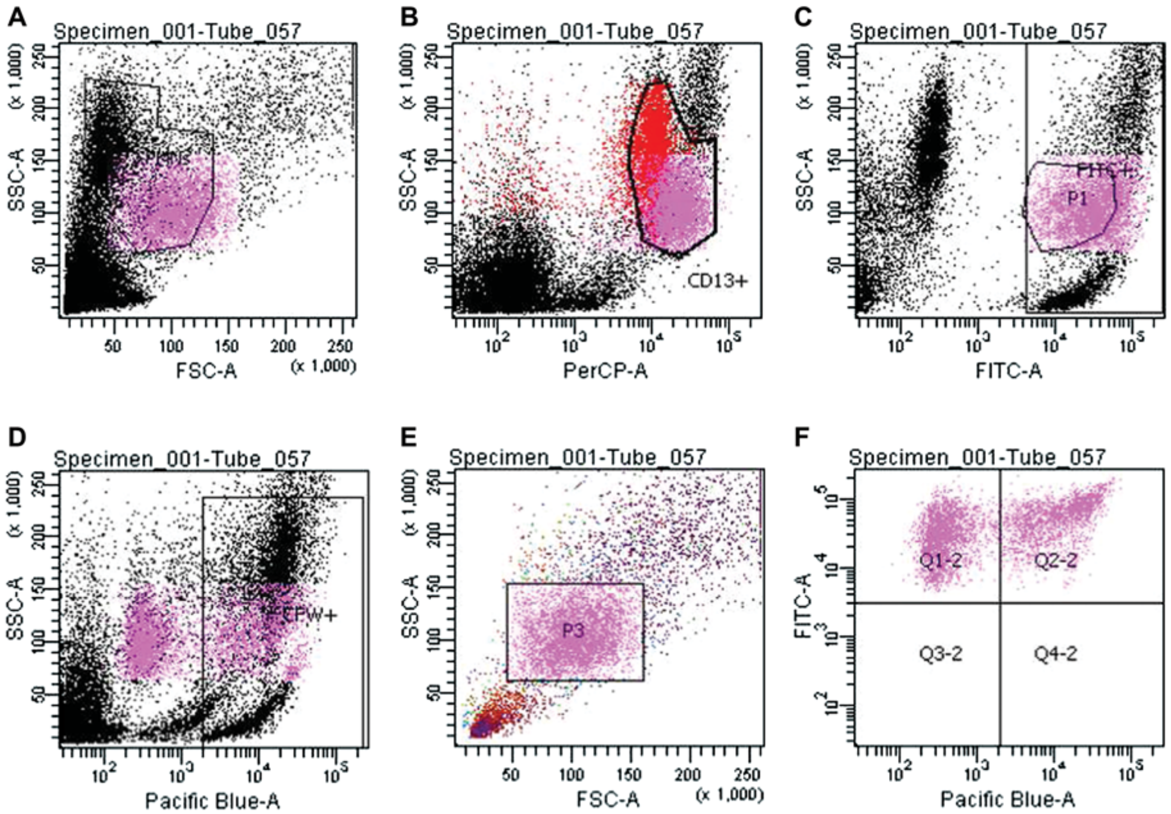

Fig. S1 Compiled panel showing the gating strategy and results of a typical FACs assay of binding and phagocytosis. (A) The PMN population is characterized based on forward scatter (FSC) and side-scatter (SSC). (B) The $CD13^{+}$ gate defines the labeled PMN population (confirmed by back-gating to the FSC vs SSC plot) by an SSC vs peridinin-chlorophyll-protein (PerCP) channel plot. (C) An SSC vs FITC channel plot of the total population defined the FITC+(labelled *C. albicans*) subgroup. (D) Total SSC was plotted against signal from the Pacific Blue channel to define events staining positive for CFW. (E) A SSC vs FSC plot of the total FITC+population revealed a cluster of events mid way along both axes. A gate was applied showing that these events were labelled PMN associating with FITC-labelled *C. albicans* cells. (F) A FITC vs Pacific Blue-channel plot of the P3 population shows two distinct sub-groups of events corresponding to bound (FITC+, Pacific Blue+) and engulfed (FITC+, Pacific Blue –) *C. albicans* cells.

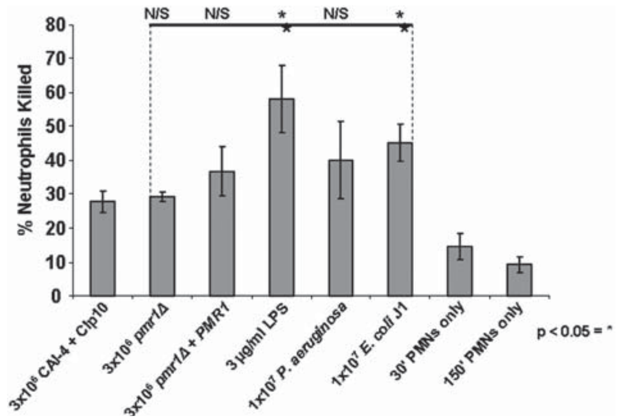

Fig. S2 Neutrophil killing following 30 min pre-stimulation with *Candida albicans*, bacteria and LPS. PMNs (10^6 cells) were stimulated by either *C. albicans* strains (3×10^6 cells), 3 µg/ml LPS, *P. aeruginosa*, or *E. coli* (heat-killed, 10^7 cells) for 30 min at 37°C. The proportion of dead PMNs following this incubation period was determined by Trypan Blue dye exclusion assay. A minimum of 100 cells were counted and the data represents the mean and standard error of three unique experiments in which triplicate measurements were made. Statistical analysis was by Student's t-tests (versus the value from PMNs stimulated by the control *C. albicans* strain, CAI-4 + CIp10 in each case): $^{**}P < 0.05$.

engulfed and attached *C. albicans* are associated with PMN cells. Data used in the final analysis was generated from Fig. S1F. The FITC- versus Pacific Blue-channels, plotting population P3 only, separating the population of adhered *C. albicans* (Q2-2, FITC+, Pacific Blue+) from engulfed *C. albicans* cells (Q1-2, FITC+, Pacific Blue –) is shown. This illustrates *C. albicans* cells associated with PMNs, and, within that population, the percentages of adhered versus engulfed *C. albicans* yeast cells (see Table S1 for raw values).

Supplementary Methods 2: Assay quantifying killing of Candida albicans cells by supernatant from stimulated neutrophils

Aliquots of 1×10^6 freshly isolated human neutrophil cells were co-incubated, for 30 min at 37°C in RPMI-1640 medium supplemented with 10% donor serum with continuous shaking, with microbial cell samples. Each individual aliquot of neutrophil cells was coincubated with 1×10^8 cells of either *Pseudomonas aeruginosa*, or

Table S1 Complete analysis of non phagocytosis-dependent killing of *Candida albicans* by PMNs. The control CAI-4 + CIp10, *pmr1*Δ, and *pmr1*Δ + *PMR1 C. albicans* strains, LPS, *Escherichia coli*, and *P. aeruginosa* were co-incubated with human PMNs at 3:1 (yeast:PMN) or 10:1 (bacteria:PMN) ratios. The percentage of dead PMNs following 30 min co-incubation at 37°C in RPMI-1640 + 10% donor's serum was enumerated by Trypan Blue dye exclusion. Subsequently supernatants from pre-stimulated PMNs were applied to the test strains CAI-4 + CIp10, *pmr1*Δ, and *pmr1*Δ + *PMR1* for 150 min at 37°C in a shaking waterbath. Final supernatant concentrations of MPO and IL-8 were quantified by ELISA.

Pre-stimulant (30')	Test strain (150')	% Dead PMN	% Dead Candida	MPO (ng/ml)	IL-8 (ng/ml)
3 × 10⁶ CAI-4 + CIp10	–	28 ± 3	–	20 ± 1	16 ± 4
3 × 10⁶ *pmr1*Δ	–	29 ± 1	–	24 ± 2	15 ± 4
3 × 10⁶ *pmr1*Δ + *PMR1*	–	37 ± 7	–	25 ± 3	16 ± 3
3 µg/ml LPS	–	58 ± 10	–	9 ± 1	16 ± 2
1 × 10⁷ *P. aeruginosa*	–	40 ± 11	–	13 ± 2	16 ± 1
1 × 10⁷ *E. coli*	–	45 ± 5	–	12 ± 4	16 ± 1
3 × 10⁶ CAI-4 + CIp10	CAI-4 + CIp10	–	29 ± 3	22 ± 3	27 ± 10
3 × 10⁶ *pmr1*Δ	CAI-4 + CIp10	–	37 ± 2	17 ± 3	18 ± 7
3 × 10⁶ *pmr1*Δ + *PMR1*	CAI-4 + CIp10	–	35 ± 1	17 ± 3	18 ± 9
3 µg/ml LPS	CAI-4 + CIp10	–	42 ± 3 (*)	6 ± 3	18 ± 7
1 × 10⁷ *P. aeruginosa*	CAI-4 + CIp10	–	43 ± 2 (*)	6 ± 3	28 ± 4
1 × 10⁷ *E. coli*	CAI-4 + CIp10	–	47 ± 4 (*)	7 ± 3	34 ± 3
3 × 10⁶ CAI-4 + CIp10	*pmr1Δ*	–	41 ± 9	16 ± 3	13 ± 2
3 × 10⁶ *pmr1*Δ	*pmr1Δ*	–	45 ± 6	24 ± 3	13 ± 7
3 × 10⁶ *pmr1*Δ + *PMR1*	*pmr1Δ*	–	42 ± 8	31 ± 3	25 ± 8
3 µg/ml LPS	*pmr1Δ*	–	45 ± 7	6 ± 3	15 ± 5
1 × 10⁷ *P. aeruginosa*	*pmr1Δ*	–	46 ± 9	8 ± 3	15 ± 5
1 × 10⁷ *E. coli*	*pmr1Δ*	–	43 ± 9	11 ± 3	22 ± 13
3 × 10⁶ CAI-4 + CIp10	*pmr1Δ + PMR1*	–	39 ± 3	25 ± 3	26 ± 9
3 × 10⁶ *pmr1*Δ	*pmr1Δ + PMR1*	–	39 ± 8	37 ± 3	24 ± 8
3 × 10⁶ *pmr1*Δ + *PMR1*	*pmr1Δ + PMR1*	–	41 ± 5	26 ± 3	23 ± 7
3 µg/ml LPS	*pmr1Δ + PMR1*	–	41 ± 3	6 ± 3	15 ± 1
1 × 10⁷ *P. aeruginosa*	*pmr1Δ + PMR1*	–	40 ± 5	16 ± 3	33 ± 8
1 × 10⁷ *E. coli*	*pmr1Δ + PMR1*	–	40 ± 2	8 ± 3	29 ± 7

Key (%)

- 0–20
- 21–40
- 41–60
- 61–80
- 81–100

Escherichia coli or 1 × 10^6 cells of either *C. albicans* CAI-4*, C. albicans pmr1*Δ *or C. albicans pmr1*Δ + *PMR1*. Following incubation, supernatants were harvested and co-incubated with 1 × 10^6 freshly harvested mid-exponential cells of either *C. albicans* CAI-4, *C. albicans pmr1*Δ *or C. albicans pmr1*Δ + *PMR1* for 150 min at 37° with shaking. Additionally, PMN viability following the pre-stimulation period was assessed by Trypan Blue dye exclusion assay [20]. Following the incubation period, fungal cells were washed three times with sterile PBS and resuspended in 50 μl of 4 μg/ml propidium iodide in PBS and visualized by microscopy. The percentages of dead cells with PI-stained nuclei were quantified by fluorescence microscopy. A minimum of three groups of 100 cells were counted for each strain and the experiment repeated for a total of six independent biological replicates.
